# Cyclic Stretch Negatively Regulates IL-1β Secretion Through the Inhibition of NLRP3 Inflammasome Activation by Attenuating the AMP Kinase Pathway

**DOI:** 10.3389/fphys.2018.00802

**Published:** 2018-06-28

**Authors:** Kentaro Maruyama, Yukihiko Sakisaka, Mizuki Suto, Hiroyuki Tada, Takashi Nakamura, Satoru Yamada, Eiji Nemoto

**Affiliations:** ^1^Department of Periodontology and Endodontology, Tohoku University Graduate School of Dentistry, Sendai, Japan; ^2^Department of Oral Immunology, Tohoku University Graduate School of Dentistry, Sendai, Japan; ^3^Department of Dental Pharmacology, Tohoku University Graduate School of Dentistry, Sendai, Japan

**Keywords:** cyclic stretch, inflammasome, macrophage, IL-1β, AMPK

## Abstract

Macrophages are immune cells of hematopoietic origin that play diverse roles in host defenses and tissue homeostasis. In mechanical microenvironments, macrophages receive mechanical signals that regulate various cellular functions. However, the mechanisms by which mechanical signals influence the phenotype and function of macrophages in the process of inflammation have not yet been elucidated in detail. We herein examined the effects of cyclic stretch (CS) on NLR family, pyrin domain-containing 3 (NLRP3) inflammasome activation in J774.1, a murine macrophage cell line, and mouse primary bone marrow-derived macrophages. We showed that cyclic stretch inhibited adenosine triphosphate (ATP)-stimulated interleukin (IL)-1β secretion in lipopolysaccharide (LPS)-primed macrophages using ELISA and Western blot analyses. Cyclic stretch did not affect the degradation of the Inhibitor of κB or the nuclear translocation/transcriptional activity of nuclear factor (NF)-κB, suggesting that cyclic stretch-mediated inhibition was independent of the NF-κB signaling pathway. Consistent with these results, cyclic stretch did not affect the LPS-induced expression of inflammasome components, such as pro-IL-1β and NLRP3, which is known to require the activation of NF-κB signaling. We showed that the cyclic stretch-mediated inhibition of IL-1β secretion was caused by the suppression of caspase-1 activity. The addition of compound C, a specific inhibitor of adenosine monophosphate-activated protein kinase (AMPK), to LPS-primed macrophages inhibited IL-1β secretion as well as caspase-1 activation, suggesting that AMPK signaling is involved in ATP-triggered IL-1β secretion. Furthermore, the phosphorylation of AMPK induced by ATP in LPS-primed macrophages was significantly suppressed by cyclic stretch, indicating that cyclic stretch negatively regulates IL-1β secretion through the inhibition of caspase-1 activity by attenuating the AMPK pathway. Our results suggest that mechanical stress functions to maintain homeostasis through the prevention of excessive inflammasome activation in macrophages in mechanical microenvironments.

## Introduction

Mechanical stress plays a critical role in the regulation of many cellular functions including cell proliferation, differentiation, and inflammation/immune responses, which are required for the maintenance of tissue homeostasis. In mechanical environments, cells sense and respond to mechanical forces, and mechanical signals are converted to chemical signals that regulate genetic and biochemical signaling pathways as a part of their physiological function (Discher et al., 2005). Cells encounter many dynamic mechanical forces, e.g., shear stress from fluid flow, mechanical loading in bone or cartilage tissue, or cyclic stretch from several tissues such as the periodontal ligament, lung, heart, and vasculature. Cells that are surrounded by an extracellular matrix (ECM) also encounter static mechanical forces with various physical properties, such as stiffness and the topography of the ECM (McWhorter et al., 2015; Mennens et al., 2017).

Macrophages play diverse roles in host defenses and tissue homeostasis by protecting the organism from infection and remodeling tissues after inflammation (Lavin et al., 2015). In adults, most tissue-specific macrophages are derived from the yolk sac during embryonic development and are maintained through self-renewal with a minimal contribution by adult circulating blood monocytes (Lavin et al., 2015). On the other hand, a large number of blood monocytes, which are derived from bone marrow, infiltrate injured/inflamed peripheral tissues and differentiate into macrophages (Papaccio, 1993; Shi and Pamer, 2011), which then receive various types of signals depending on the microenvironments and play a central role in the processes of inflammation and tissue repair. However, the mechanisms by which mechanical signals influence the phenotype and function of macrophages during this series of processes remain unclear.

Inflammasome activation represents inflammatory responses during bacterial infection or tissue injury, which are required for the clearance of pathogens or repair of injured tissues. However, sustained or excessive inflammasome activation may exacerbate pathological inflammation (Davis et al., 2011). Inflammasomes are a group of cytosolic protein complexes that regulate the activation of caspase-1, which leads to the processing of pro-interleukin (IL)-1β and pro-IL-18 to their mature active forms, as well as pyroptosis, an inflammatory programmed cell death (Martinon et al., 2002). A number of nucleotide-binding oligomerization domain (NOD)-like receptor (NLR) family members have been shown to form inflammasomes in response to various stimuli. Among them, NLR family, pyrin domain-containing 3 (NLRP3) has been characterized in the most detail because it responds to various stimuli including pathogen-associated molecules and endogenous damage/danger-associated molecular patterns, such as extracellular adenosine triphosphate (ATP), urate crystals, β-amyloids, and cholesterol crystals (Mariathasan et al., 2006; Martinon et al., 2006; Halle et al., 2008; Duewell et al., 2010). The NLRP3 inflammasome consists of the NLRP3 protein, the adaptor protein apoptotic speck-like protein containing a caspase recruitment domain (ASC), and caspase-1. The activation of the NLRP3 inflammasome is a two-step process: the expression of NLRP3 and pro-IL-1β is induced by transcriptional up-regulation via nuclear factor (NF)-κB signaling (Bauernfeind et al., 2009), and NLRP3 inflammasome protein components are then assembled after exposure to microbial pathogens and endogenous danger signals in order to form a platform to activate caspase-1, which cleaves pro-IL-1β and pro-IL-18, thereby allowing it to be secreted from cells (Martinon et al., 2002).

Since limited information is currently available on the relationship between inflammasome signaling and the mechanical environment, we investigated whether mechanical signals from cyclic stretch regulate NLRP3 inflammasome signaling in murine monocyte-derived macrophages. The present results demonstrated that cyclic stretch significantly inhibited ATP-triggered IL-1β secretion as well as pyroptosis in lipopolysaccharide (LPS)-primed macrophages independently of the NF-κB signaling pathway, and this inhibition was partially due to the suppression of 5′ adenosine monophosphate (AMP)-activated protein kinase (AMPK) activity.

## Materials and Methods

### Reagents

ATP disodium salt, LPS (*Escherichia coli* O55:B5), and dimethyl sulfoxide (DMSO) were purchased from Sigma (St. Louis, MO, United States). Recombinant mouse macrophage-colony stimulating factor (rmM-CSF) was purchased from PeproTech Inc. (Rocky Hill, NJ, United States). Compound C was purchased from Merck Millipore (Darmstadt, Germany).

### Cell Line and Cell Culture

The mouse macrophage-like cell line J774.1 was obtained from the Cell Resource Center for Biomedical Research, the Institute of Development, Aging, and Cancer, Tohoku University, and cultured in RPMI1640 (Gibco BRL, Rockville, MD, United States) with 10% heat-inactivated fetal bovine serum (FBS) (Gibco BRL) and antibiotics (100 U/ml penicillin G and 100 μg/ml streptomycin) in a controlled (5% CO_2_) humidified atmosphere.

### Bone Marrow-Derived Macrophages (BMDM)

Bone marrow cells were harvested from the femurs of 6-week-old C57BL/6 mice (Kumagai-shigeyasu Co., Ltd., Miyagi, Japan), treated with 1 × RBC Lysis Buffer^®^ (Affymetrix, San Diego, CA, United States), and cultured at 5 × 10^5^ cells/ml in DMEM/F12 (Gibco BRL) with 10% FBS and antibiotics in the presence of 10 ng/ml rmM-CSF in a controlled (5% CO_2_) humidified atmosphere. After being cultured for 3 and 5 days, fresh medium was added. After 7 days of culture, unattached cells were removed and attached cells were used in experiments.

### Application of Cyclic Stretch

A uniaxial cyclic tensile force was applied to cells with a STB-140 STREX cell stretch system (STREX Co., Osaka, Japan). Regarding the collagen coating of a silicon resin chamber (STB-CH-10.0, STREX Co.), 2 ml of 300 μg/ml type I atelo-collagen (Atelo Cell^®^, KOKEN Co., Tokyo, Japan) was added to chambers and dried overnight on a clean bench. J774.1 cells were seeded at 1.5 × 10^6^ cells or BMDM at 0.5 × 10^6^ cells on a collagen-coated chamber with an area of 10.24 cm^2^, and then cultured for 24 h in medium with 10% FBS. After 24 h, medium was exchanged with that containing 5% FBS, and cells were exposed to cyclic stretch. Cells were maintained in a controlled (5% CO_2_) humidified atmosphere.

### Enzyme-Linked Immunosorbent Assay (ELISA) for IL-1β

Supernatants from cell cultures were harvested by centrifugation. The amount of IL-1β in the supernatants was measured using the IL-1β Mouse SimpleStep ELISA^TM^ Kit (ab197742, Abcam plc, Cambridge, United Kingdom). The amount of IL-1β was measured using the Softmax data analysis program (Molecular Devices, Menlo Park, CA, United States).

### Reverse Transcription and Real-Time Quantitative Polymerase Chain Reaction (RT-PCR)

Total RNA was extracted using Qiashredder and RNeasy^®^ Kits (QIAGEN, Valencia, CA, United States) according to the manufacturer’s instructions, and then treated with DNase (DNA-free^TM^, Ambion Inc., Austin, TX, United States). Total RNA was reverse-transcribed using a Transcriptor First Strand complementary DNA (cDNA) Synthesis Kit^®^ (Roche Diagnostic Co., Indianapolis, IN, United States) according to the manufacturer’s instructions. cDNA, theoretically converted from 50 ng of total RNA, was used. In real-time PCR, the amplification profile was 40 cycles at 95/10, 55/30, and 72/30 [temperature (°C)/time (sec)]. PCR was performed using the CFX96 Touch^TM^ Real-Time PCR Detection System (Bio-Rad Laboratories, Hercules, CA, United States) with iQ SYBR Green Supermix^®^ (Bio-Rad Laboratories) and optimized levels of 3 mM MgCl_2_ and 500 nM of each primer. The relative expression levels of transcripts are shown after normalization to the corresponding sample expression level of glyceraldehyde 3-phosphate dehydrogenase (GAPDH). Primer sequences for each mouse gene encoding IL-1β, tumor necrosis factor-α (TNF-α), cyclooxygenase-2 (COX-2), NLRP3, IL-6, and GAPDH were as follows (forward/reverse): mouse *Il-1*β (5′-ATGGGCAACCACTTACC-3′/5′-AATGAAAGACCTCAGTGCG-3′); mouse *Tnf*-α (5′-GCTTTCAACAACTACTCAGAAAC-3′/5′-GACCACTCTCCCTTTGC-3′); mouse *Cox-2* (5′-ACAACAGAGTGTGCGAC-3′/5′-TGAGTTTGAAGTGGTAACCG-3′); mouse *Nlrp3* (5′-GACTCAGGCTCCTCTGTG-3′/5′-CTACCAGGAAATCTCGAAGAC-3′); mouse *Il-6* (5′-ACTGATGCTGGTGACA-3′/5′-GCAAGTGCATCATCGT-3′); mouse *Gapdh* (5′-ACCACAGTCCATGCCATCAC-3′/5′-TCCACCACCCTGTTGCTGTA-3′).

### Western Blotting

Cells were harvested with Cell Lysis Buffer^®^ (Cell Signaling Technology, Beverly, MA, United States) in accordance with the manufacturer’s instruction. Cell supernatants were centrifuged using Amicon^®^ Ultra-2 (nominal molecular weight limit: 50 kDa) (Merck Millipore, Darmstadt, Germany) to remove the albumin fraction, and concentrated 20 times using StrataClean^TM^ resin (Agilent, Santa Clara, CA, United States). Protein samples were treated with 2 × Laemmli sample buffer (Bio-Rad Laboratories), separated by sodium dodecyl sulfate-polyacrylamide gel electrophoresis, and transferred to a polyvinylidene difluoride membrane (ATTO, Tokyo, Japan) using a semidry transblot system (ATTO). The blot was blocked with 0.5% (w/v) non-fat skim milk and 0.1% (v/v) Tween 20 in phosphate-buffered saline (PBS) at room temperature for 1 h, followed by an incubation at room temperature for 1 h with a rabbit anti-inhibitor of κB (IκB)-α (Cell Signaling Technology), anti-pro Caspase1+p10+p12 (ab179515, Abcam), anti-IL-1β (D6D6T), phosphor-AMPKα (Thr172) (40H9, Cell Signaling Technology), AMPKα (D63G4, Cell Signaling Technology), anti-IL-6 (D5W4V, Cell Signaling Technology), anti-TNF-α (D2D4, Cell Signaling Technology), anti-NLRP3 (D4D8T, Cell Signaling Technology), and mouse-anti-P2X7 (D-1, Santa Cruz Biotechnology, Dallas, TX, United States) at 1:1000. Blots were incubated with horseradish peroxidase (HRP)-conjugated goat anti-rabbit IgG (Cell Signaling Technology) or HRP-conjugated goat anti-mouse IgG (Cell Signaling Technology) at 1:2000 at room temperature for 1 h. Blots was then treated with the Western blotting detection reagent ECL Plus^®^ (Amersham Pharmacia Biotech Inc., Piscataway, NJ, United States), and chemiluminescent signals were detected using the luminescent image analyzer, ChemiDoc XRS Plus^TM^ (Bio-Rad Laboratories).

### Immunocytochemistry Assay

Cells were fixed with ice cold 100% methanol at −20°C for 30 min, incubated with PBS containing 0.25% Triton X-100 (PBS-T) for 10 min to permeabilize cells, and then incubated in PBS-T containing 1% (w/v) bovine serum albumin (Sigma) for 30 min to block non-specific binding. Cells were then incubated with a 1:1000 dilution of a rabbit anti-NF-κB p65 monoclonal antibody (D14E12, Cell Signaling Technology) for 1 h followed by a 1:1000 dilution of an Alexa Fluor^®^ 488 conjugated-goat anti-rabbit secondary antibody (Invitrogen, Carlsbad, CA, United States) for 1 h. After being incubated with 4′,6-diamidino-2-phenylindole (DAPI) (Invitrogen) for 1 min to identify nuclei, staining was evaluated by immunofluorescence microscopy. In a quantitative analysis, cells showing the nuclear translocation of NF-κB p65 in three randomly selected fields (each containing ∼100 cells) were quantified.

### NF-κB p65 ELISA

Nuclear proteins were extracted using a Nuclear Extract Kit (Active Motif, Carlsbad, CA, United States) according to the manufacturer’s instructions. Activated NF-κB of extracted nuclear proteins (10 μg/well) was measured with TransAM ^®^NF-κB p50, p52, p65, and Family Kits (Active Motif) according to the manufacturer’s instructions. The value of optical density at 450 nm was assessed using the Softmax data analysis program (Molecular Devices). Nuclear proteins from Raji cells (5 μg/well) were used as a positive control, and a sample with no cell extract was used as a negative control.

### Whole-Cell Caspase-1 Activity

Whole-cell caspase-1 activity was detected using FAM-FLICA^®^ caspase-1 assay kits (Immunochemistry Technologies, Bloomington, MN, United States) according to the manufacturer’s instructions. Briefly, cells were incubated with medium containing carboxyfluorescein (FAM)-labeled fluorescent-labeled inhibitor of caspase (FLICA) for 30 min, followed by an incubation with Hoechst 33342 for 5 min to identify nuclei. Caspase-1-active cells were detected by immunofluorescence microscopy. Cells expressing active caspase-1 in three randomly selected fields (each containing ∼100 cells) were quantified.

### Lactate Dehydrogenase (LDH) Analysis

Lactate Dehydrogenase activity in supernatants was measured using Cytotoxicity LDH Assay Kit-WST (Dojindo, Kumamoto, Japan) according to the manufacturer’s instructions. LDH from cells incubated with 10% (v/v) Lysis Buffer (Dojindo) was used as a positive control. The value of optical density at 490 nm was assessed using the Softmax data analysis program (Molecular Devices). Released LDH was calculated as follows: [(Experimental OD_490_-background OD_490_)/(positive control OD_490_-background OD_490_)] × 100(%).

### Detection of Pyroptosis

Pyroptosis was detected by propidium iodide (PI) staining (Immunochemistry Technologies). Briefly, cells were incubated with 0.5% (v/v) PI for 5 min, and with Hoechst 33342 for 5 min to identify nuclei. PI-positive cells were detected by immunofluorescence microscopy. Cells stained by PI in three randomly selected fields (each containing ∼100 cells) were quantified.

### Statistical Analysis

All experiments in the present study were performed three times to test the reproducibility of the results, and representative findings are shown. Experimental values are given as means ± *SD*. The significance of differences between control and treatment experiments was evaluated by the Kruskal-Wallis test with the Steel *post hoc* test. *P* values < 0.05 were considered to be significant.

## Results

### Cyclic Stretch Inhibits ATP-Stimulated IL-1β Secretion in LPS-Primed Macrophages

**FIGURE 1:**
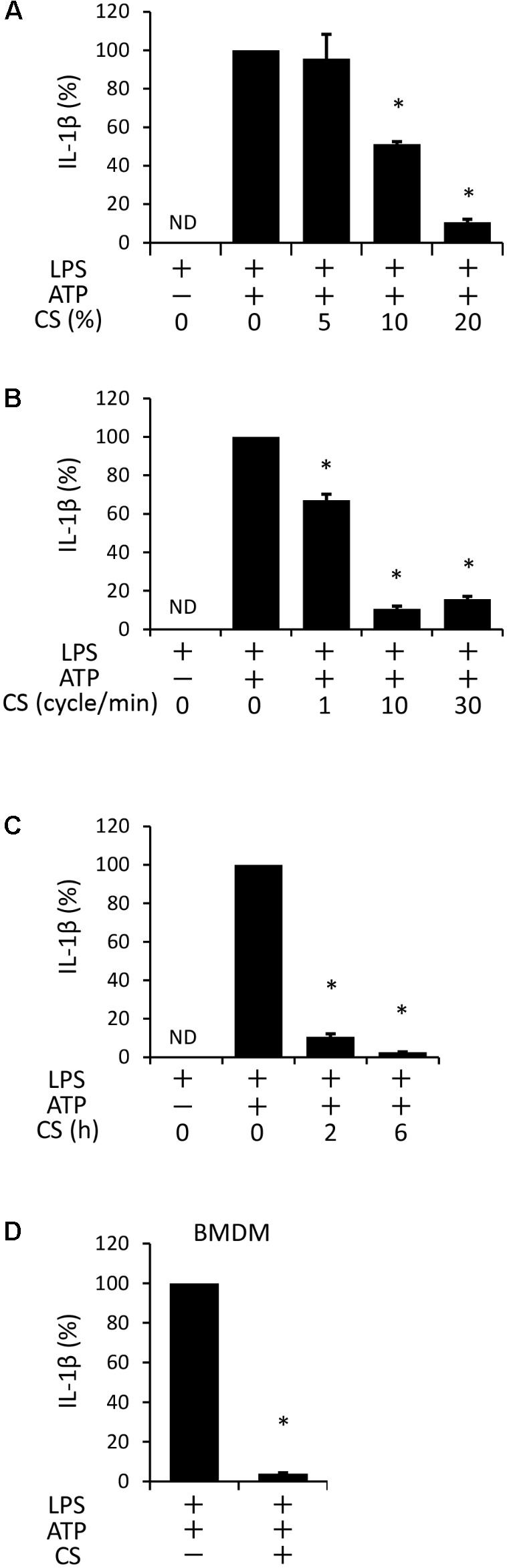
Cyclic stretch (CS) inhibits adenosine triphosphate (ATP)-stimulated IL-1β secretion in lipopolysaccharide (LPS)-primed macrophages. The murine macrophage cell line J774.1 **(A–C)** and murine Bone Marrow-Derived Macrophages (BMDM) **(D)** were primed with 100 ng/ml of *Escherichia coli* LPS for 4 h followed by a stimulation with 1 mM ATP for 2 h in the continuous presence of LPS. Cells were exposed to various conditions of CS as follows during the LPS stimulation. **(A)**: J774.1 cells were exposed to CS of 5, 10, and 20% elongation at a frequency of 10 cycles/min for the first 2 h after the addition of LPS. **(B)**: J774.1 cells were exposed to CS of 1, 10, or 30 cycles/min at a constant rate of 20% elongation for the first 2 h after the addition of 100 ng/ml LPS. **(C)**: J774.1 cells were exposed to CS of 20% elongation at a frequency of 10 cycles/min for the first 2 or 6 h after the addition of LPS. **(D)**: BMDM were exposed to CS of 20% elongation at a frequency of 10 cycles/min for the first 2 h after the addition of LPS. **(A–D)**: The amount of IL-1β in the supernatant was analyzed by Enzyme-Linked Immunosorbent Assay (ELISA). Representative data of three separate experiments are shown as the means ± *SD* of triplicate assays. Significance is indicated (^∗^*P* < 0.05 significantly different from the positive control).

We examined the effects of cyclic stretch on ATP-stimulated IL-1β secretion in the LPS-primed macrophage cell line, J774.1. Cells were primed with 100 ng/ml of *E. coli* LPS for 4 h followed by a stimulation with 1 mM ATP, an NLRP3 inflammasome activator, for 2 h in the continuous presence of LPS. The amount of IL-1β in the supernatant was measured by ELISA. A significant amount of IL-1β was detected and its concentration was 265.1 ± 73 pg/ml, which was converted to 100% as IL-1β secretion (**Figure 1A**, the second bar from the left). When cells were exposed to cyclic stretch at a constant frequency of 10 cycles/min for the first 2 h after the addition of LPS, a significant decrease in IL-1β secretion was detected at 10% elongation, while further inhibition (89 ± 1.5% inhibition) was observed at 20% elongation (**Figure 1A**). When cells were exposed to cyclic stretch at 1, 10, or 30 cycles/min at 20% elongation, the decrease in IL-1β secretion almost plateaued at 10 cycles/min (**Figure 1B**). In order to examine the effects of the duration of cyclic stretch on IL-1β secretion, cells were exposed to cyclic stretch at 20% elongation and at 10 cycles/min for the first 2 or 6 h after the addition of LPS. **Figure 1C** shows marked decreases in IL-1β secretion under all duration conditions. Based on these results, subsequent experiments were performed under the condition of 20% elongation, 10 cycles/min, and the first 2-h exposure during LPS priming. We then evaluated whether the inhibition of IL-1β was reproduced in murine primary macrophages, BMDM. **Figure 1D** shows that the ATP stimulation induced IL-1β secretion from LPS-primed BMDM at a concentration of 104.1 ± 25 pg/ml, which was converted to 100% as IL-1β secretion in **Figure 1D** (left bar). The value of secreted IL-1β was similar to that in J774.1 cells in terms of per cell number. Cyclic stretch significantly inhibited IL-1β secretion to a similar level to that in J774.1 cells. We also found that cyclic stretch-mediated inhibition was selective for IL-1β, but not for other inflammatory molecules, such as IL-6, TNF-α, and Cox-2, as assessed by real-time PCR (Supplemental Figures S1A–C) and a Western blot analysis (Supplemental Figure S1D).

### Cyclic Stretch Does Not Alter the LPS-Induced NF-κB Signaling Pathway

**FIGURE 2:**
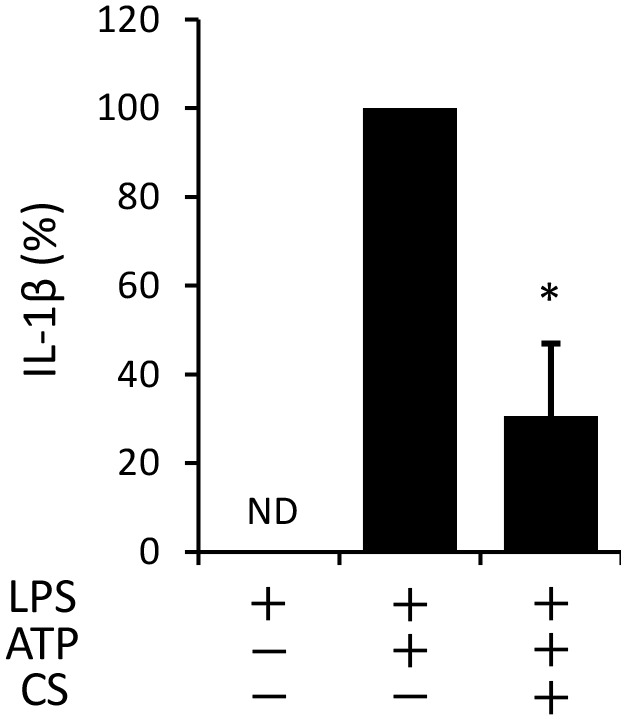
Cyclic stretch exerts direct inhibitory effects on ATP triggering in LPS-primed macrophages. J774.1 cells were primed with 100 ng/ml of *E. coli* LPS for 4 h and exposed to CS of 20% elongation at a frequency of 10 cycles/min just before the addition of 1 mM ATP for 2 h. The amount of IL-1β in the supernatant was analyzed by ELISA. Representative data of three separate experiments are shown as the means ± *SD* of triplicate assays. Significance is indicated (^∗^*P* < 0.05 significantly different from the positive control).

**FIGURE 3:**
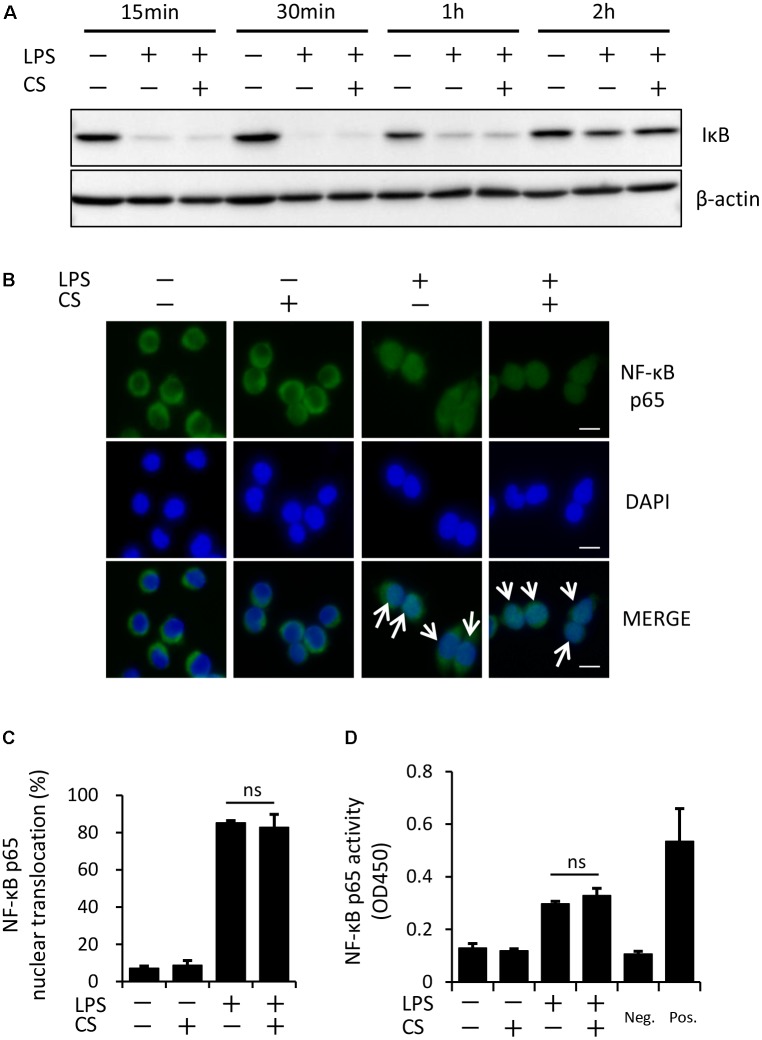
Cyclic stretch does not alter the LPS-induced NF-κB signaling pathway. **(A)**: J774.1 cells were exposed to CS of 20% elongation at a frequency of 10 cycles/min with 100 ng/ml LPS for the indicated times. Cell lysates were analyzed by Western blotting with anti-IκB-α. An antibody against β-actin was used as a control. Results are representative of three independent experiments. **(B–D)**: J774.1 cells were exposed to CS of 20% elongation at a frequency of 10 cycles/min for the first 2 h during treatment with 100 ng/ml LPS for 4 h. **(B)**: The nuclear translocation of NF-κB p65 was detected by immunostaining (green: shown in the upper panel). Nuclei were visualized by staining with DAPI (blue: shown in the middle panel). Merged images are shown in the lower panel (magnification: × 400; scale bars are 20 μm). White arrows indicate p65 nuclear translocation. Results are representative of three independent experiments. **(C)**: Cells showing the nuclear translocation of NF-κB p65 in three randomly selected fields (each containing ∼100 cells) were quantified. Representative data of three separate experiments are shown as the means ± *SD* of triplicate assays (ns; not significant). **(D)**: Nuclear proteins were extracted from cells and a NF-κB ELISA assay was performed. The positive control (Pos.) was provided by a nuclear extract (5 μg) of Raji cells. A sample with no cell extract was used as a negative control (Neg.). Representative data of three separate experiments are shown as the means ± *SD* of triplicate assays (ns; not significant).

**FIGURE 4:**
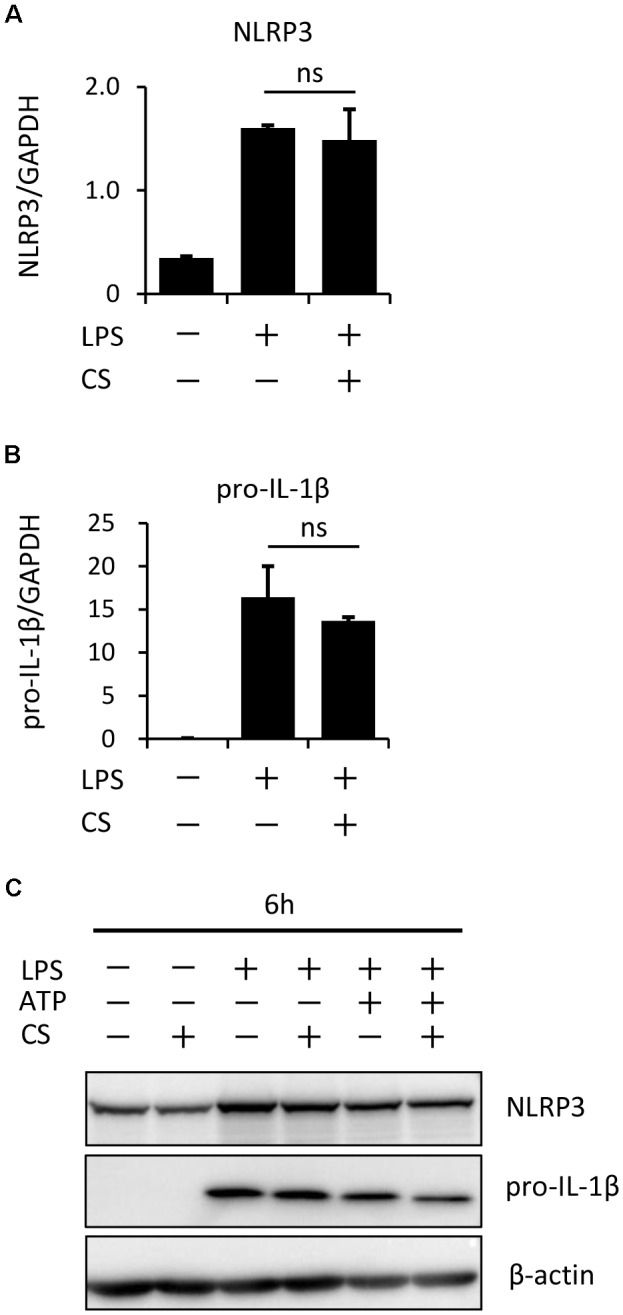
Cyclic stretch does not affect expression levels of inflammasome components. J774.1 cells were exposed to CS of 20% elongation at a frequency of 10 cycles/min for the first 2 h during a treatment with 100 ng/ml LPS for 4 h **(A,B)**, followed by a stimulation with ATP for 2 h in the continuous presence of LPS **(C)**. **(A,B)**: Total cellular RNA was extracted, and transcripts of NLRP3 and pro-IL-1β were analyzed by real-time quantitative PCR. Representative data of three separate experiments are shown as the means ± *SD* of triplicate assays (ns; not significant). **(C)**: Cell lysates were analyzed by Western blotting with anti-NLRP3, pro-IL-1β antibodies (molecular masses, NLRP3: 110 kDa, pro-IL-1β: 31 kDa). An antibody against β-actin was used as a control. Results are representative of three independent experiments.

The process of IL-1β secretion involves two phases: LPS priming and ATP triggering (Martinon et al., 2002). In order to identify which phase(s) are affected by cyclic stretch, LPS-primed macrophages were exposed to cyclic stretch just before the addition of ATP. As shown in **Figure 2**, cyclic stretch significantly inhibited IL-1β secretion, suggesting that cyclic stretch exerts direct inhibitory effects on the ATP-triggering phase. On the other hand, during the LPS priming of macrophages, activation of the NF-κB signaling pathway is known to be required for the expression of inflammasome component molecules such as pro-IL-1β and NLRP3 (Lamkanfi and Dixit, 2014). In a steady state, the NF-κB dimmer p50/p65 is maintained in the cytoplasm with binding to its inhibitory protein, IκB. The degradation of IκB upon activation with LPS results in the liberation of NF-κB, which translocates to the nucleus, in which it binds to specific response sequences on the promoters of NF-κB downstream target genes (Hayden and Ghosh, 2008). We examined whether cyclic stretch regulates the NF-κB signaling pathway during LPS priming. **Figure 3A** shows that IκB was degraded at a peak of 30 min after the LPS stimulation and was re-expressed 2 h after the LPS stimulation to a similar level of that of the control. In contrast, cyclic stretch did not affect the LPS-induced degradation or re-expression of IκB. Furthermore, cyclic stretch did not affect the LPS-induced nuclear translocation of NF-κB, which was assessed by immunocytohistochemistry, as shown in **Figures 3B,C**. We also measured NF-κB p65 DNA-binding activity to specific DNA sequences by ELISA using nuclear extracts. **Figure 3D** shows that cyclic stretch did not significantly affect LPS-induced NF-κB binding activity. Therefore, cyclic stretch appears to inhibit the ATP-triggering phase in IL-1β secretion independently of the NF-κB signaling pathway. Consistent with these results, cyclic stretch did not significantly affect the LPS-induced expression of inflammasome components, such as NLRP3 and pro-IL-1β, at the gene (**Figures 4A,B**) or protein level (**Figure 4C**), which is known to require the activation of the NF-κB signaling pathway (Bauernfeind et al., 2009).

### Cyclic Stretch Inhibits the ATP-Induced Activation of Caspase-1 in LPS-Primed Macrophages

**FIGURE 5:**
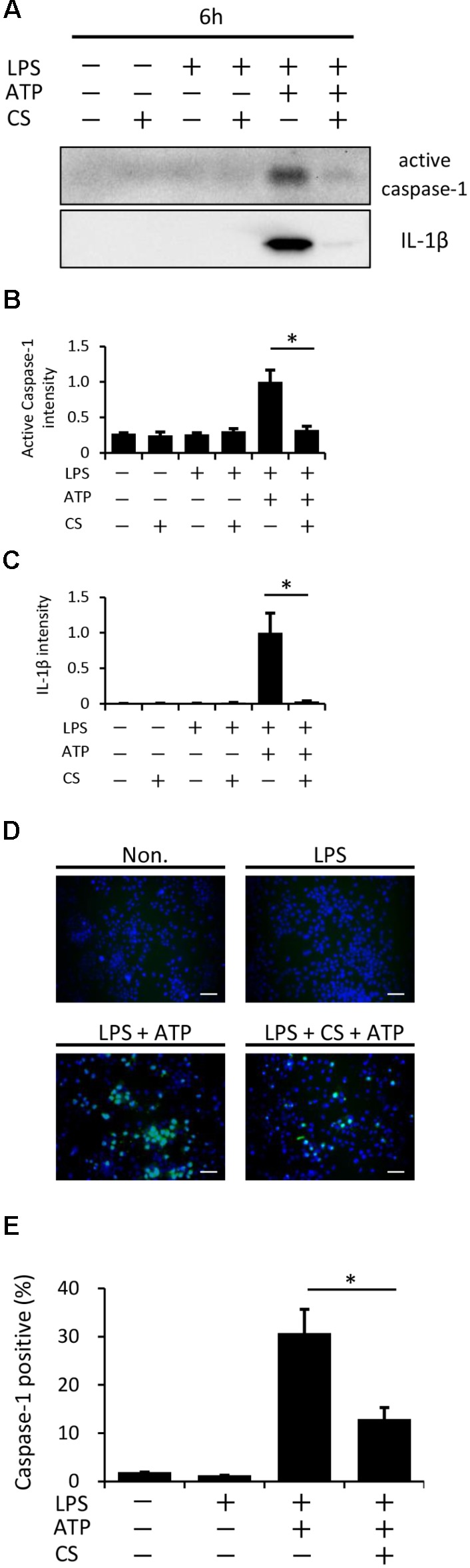
Cyclic stretch inhibits the LPS/ATP-induced activation of caspase-1. J774.1 cells were exposed to CS of 20% elongation at a frequency of 10 cycles/min for the first 2 h during a treatment with 100 ng/ml LPS for 4 h, followed by a stimulation with ATP for 2 h in the continuous presence of LPS. **(A)**: Concentrated supernatants were analyzed by Western blotting with specific antibodies to the caspase-1 and IL-1β. Molecular masses, IL-1β: 17 kDa, caspase-1: 10 kDa. **(B,C)**: The expression levels of IL-1β and caspase-1 was quantified via densitometry scanning. Results are representative of three independent experiments. **(D)**: Cells were labeled with a fluorescent-labeled inhibitor of caspase (FLICA) probe conjugated with FAM with the indication of green fluorescence and nuclei were visualized by staining with Hoechst 33342 (blue) (magnification: × 200; scale bars are 50 μm). The negative control (Non) was not treated with LPS, ATP, or CS. Results are representative of three independent experiments. **(E)**: Cells expressing active caspase-1 in three randomly selected fields (each containing ∼100 cells) were quantified. Representative data of three separate experiments are shown as the means ± *SD* of triplicate assays. Significance is indicated (^∗^*P* < 0.05 significantly different from the positive control).

A previous study reported that ATP activates NLRP3 inflammasome signaling by binding to P2X7 cell membrane receptors in LPS-primed macrophages (Gombault et al., 2013). We found that cyclic stretch did not change the expression levels of P2X7 between the unstimulated and LPS-stimulated groups, as assessed by a Western blot analysis (Supplemental Figure S2). We then investigated whether cyclic stretch affects the ATP-stimulated activation of caspase-1 in LPS-primed macrophages. As shown in **Figures 5A–C**, a stimulation with LPS/ATP induced the 10-kDa active form of caspase-1 as well as the 17-kDa active form of IL-1β in the concentrated supernatant, as assessed by the Western blot analysis, while cyclic stretch significantly reduced the expression of caspase-1 as well as IL-1β. Furthermore, we measured the expression of the active form of caspase-1 in the cytoplasm using the FLICA probe conjugated with FAM with the indication of green fluorescence. **Figures 5D,E** show that the stimulation with LPS/ATP increased the number of cells expressing the active form of caspase-1, and this increase was significantly inhibited by cyclic stretch, suggesting that the cyclic stretch-mediated inhibition of IL-1β was caused by the suppression of caspase-1 activity downstream of P2X7 receptor signaling.

### Cyclic Stretch Inhibits ATP-Induced Pyroptosis in LPS-Primed Macrophages

**FIGURE 6:**
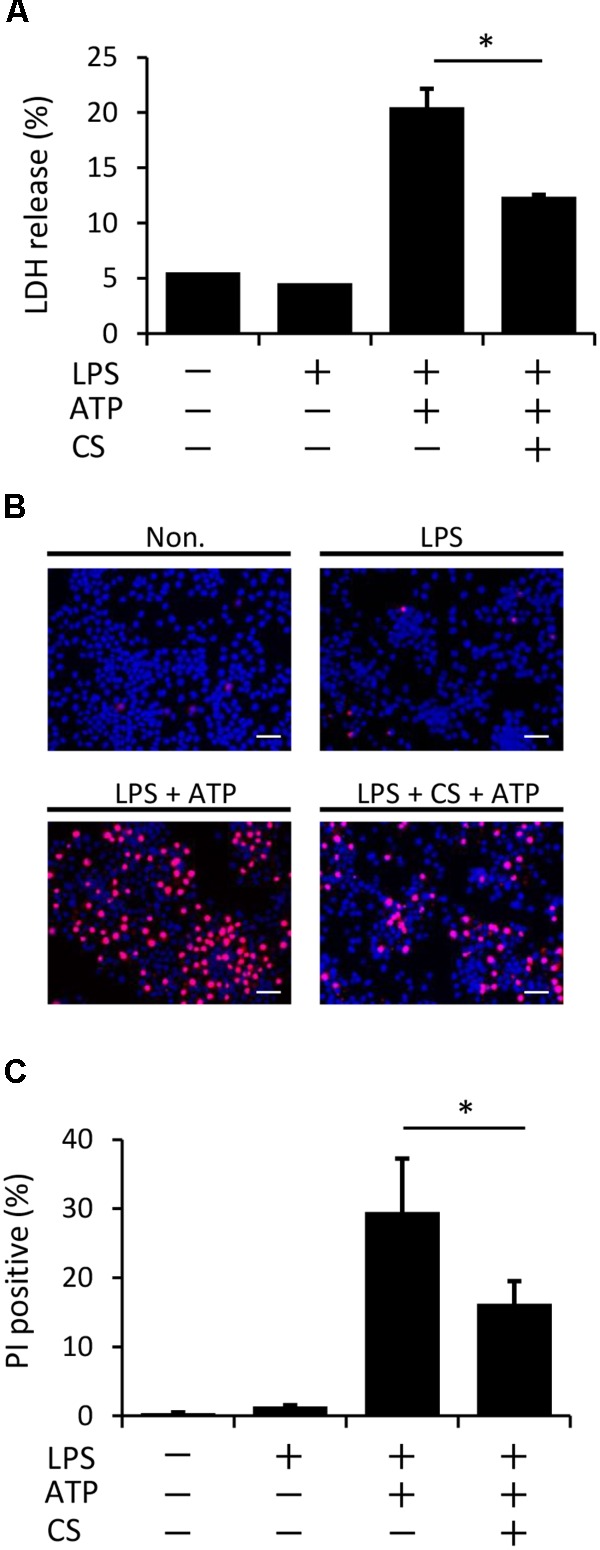
Cyclic stretch inhibits LPS/ATP-induced pyroptosis. J774.1 cells were exposed to CS of 20% elongation at a frequency of 10 cycles/min for the first 2 h during a treatment with 100 ng/ml LPS for 4 h, followed by a stimulation with ATP for 2 h in the continuous presence of LPS. **(A)**: Lactate Dehydrogenase (LDH) activity in supernatants was measured using a LDH assay kit at 490 nm. Representative data of three separate experiments are shown as the means ± *SD* of triplicate assays. **(B)**: Cells were labeled with PI with the indication of red fluorescence and nuclei were visualized by staining with Hoechst 33342 (blue) (magnification: × 200; scale bars are 50 μm). The negative control (Non) was not treated with LPS, ATP, or CS. Results are representative of three independent experiments. **(C)**: Cells stained by PI in three randomly selected fields (each containing ∼100 cells) were quantified. Representative data of three separate experiments are shown as the means ± *SD* of triplicate assays. Significance is indicated (^∗^*P* < 0.05 significantly different from the positive control).

Pyroptosis, which is mediated by caspase-1, is a type of programmed cell death with plasma membrane rupture that occurs following the activation of inflammasomes (Papaccio et al., 2005; Miao et al., 2011). We examined whether cyclic stretch inhibits ATP-induced pyroptosis in LPS-primed macrophages, as assessed by the measurement of LDH activity in the supernatant. **Figure 6A** shows that LDH activity was stronger following the stimulation with ATP/LPS-stimulated macrophages than in the unstimulated control or with LPS alone (LDH release: less than 5% in both groups), while cyclic stretch significantly inhibited the release of LDH. Furthermore, **Figures 6B,C** show that the ATP/LPS stimulation increased the number of cells stained by PI, which is commonly used to detect dead cells, while cyclic stretch reduced the number of PI-stained cells, suggesting that it inhibits ATP-induced pyroptosis in LPS-primed macrophages.

### Cyclic Stretch Inhibits ATP-Induced AMPK Activation in LPS-Primed Macrophages

**FIGURE 7:**
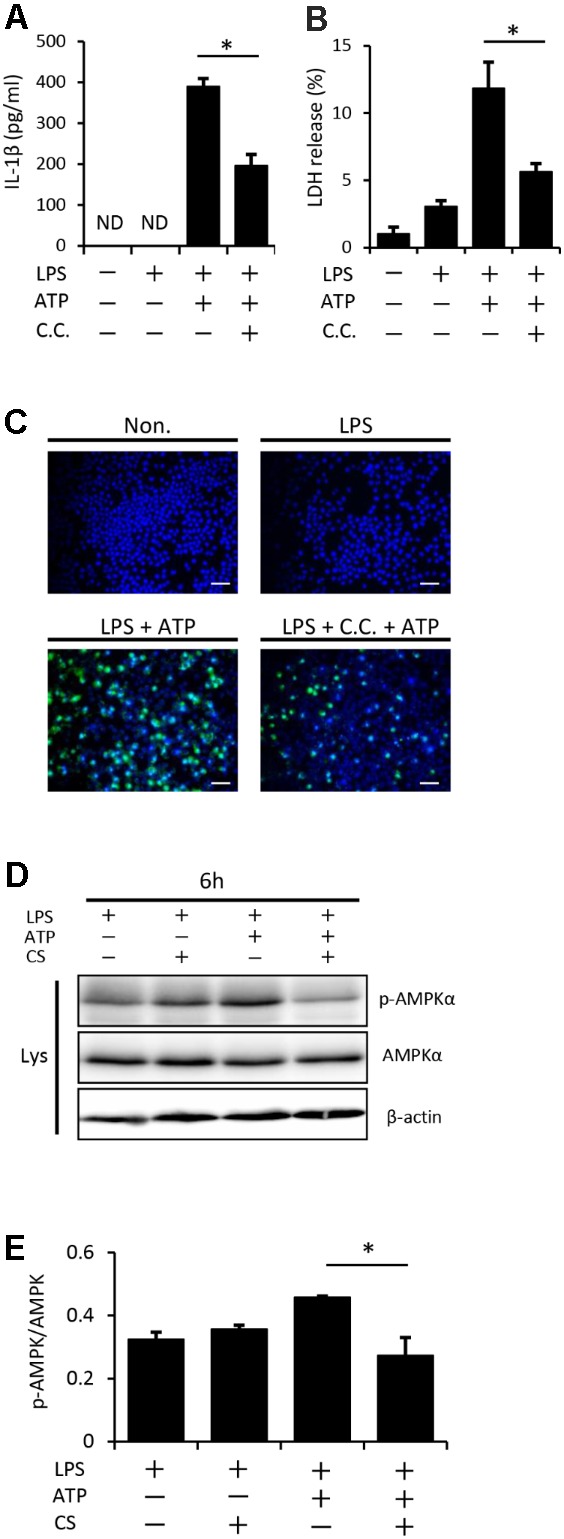
Cyclic stretch inhibits NLRP3 inflammasome activation and is partially dependent on the adenosine monophosphate-activated protein kinase (AMPK) signaling pathway. J774.1 cells were primed with 100 ng/ml LPS for 4 h, treated with 20 μM of compound C (C.C.) for 30 min, and then stimulated with 1 mM ATP for 2 h in the continuous presence of LPS and compound C **(A–C)**. All samples, including the control, were adjusted to contain 0.1% (v/v) DMSO in the culture medium during the cell culture. **(A)**: The amounts of IL-1β in the supernatant were analyzed by ELISA. **(B)**: LDH activity in supernatants was measured by a LDH assay kit at 490 nm. **(C)**: Cells were labeled with a FLICA probe conjugated with FAM with the indication of green fluorescence and nuclei were visualized by staining with Hoechst 33342 (blue) (magnification: × 200; scale bars are 50 μm). **(D,E)**: J774.1 cells were exposed to CS of 20% elongation at a frequency of 10 cycles/min for the first 2 h during a treatment with 100 ng/ml LPS for 4 h, followed by a stimulation with ATP for 2 h in the continuous presence of LPS. **(D)**: Cell lysates were analyzed by Western blotting with phosphorylated-AMPKα and AMPKα antibodies. An antibody against β-actin was used as a control (molecular mass, phosphorylated-AMPKα and AMPKα: 62 kDa). **(E)**: The expression level of phosphorylated-AMPKα was quantified via densitometry scanning. Relative expression levels of phosphorylated-AMPKα were normalized to AMPKα. Representative results of three independent experiments are shown. Significance is indicated (^∗^*P* < 0.05 significantly different from the positive control).

Previous studies reported that AMPK signaling was activated by a treatment with ATP through its binding to P2X7 receptors and has been shown to play a role in inflammasome activation (Liang et al., 2016; Zha et al., 2016). We initially investigated the possible involvement of AMPK in our experimental system using a pharmacological inhibitor. **Figure 7A** shows that the pretreatment of LPS-primed macrophages with compound C, a specific inhibitor of AMPK, followed by the addition of ATP partially inhibited inflammasome activation, such as the secretion of IL-1β (**Figure 7A**), release of LDH (**Figure 7B**), and increase in the number of cells expressing the active form of caspase-1 (**Figure 7C**), indicating that the AMPK signaling pathway is involved in LPS/ATP-induced inflammasome activation in macrophages. We then examined the effects of cyclic stretch on AMPK activity, which may be evaluated by phosphorylation at Thr172 of AMPKα, the kinase subunit of the AMPK heterotrimeric complex (Hawley et al., 1996). **Figures 7D,E** show that a certain amount of AMPKα phosphorylation observed in LPS-primed macrophages was significantly enhanced by the treatment with ATP. When cells were exposed to cyclic stretch, the phosphorylated status was significantly reduced, suggesting that the cyclic stretch-mediated inhibition of IL-1β secretion was partially due to the inhibition of AMPK signaling.

## Discussion

**FIGURE 8:**
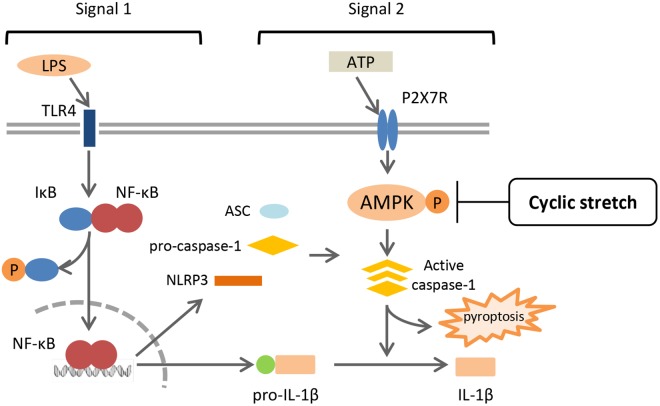
Summary of mechanisms by which CS negatively regulates IL-1β secretion in murine macrophages. A treatment with LPS activates NF-κB signaling via TLR4 (Signal 1), and induces the expression of NLRP3 and pro-IL-1β. NLRP3 inflammasome components, which consist of NLRP3, ASC, and caspase-1, are assembled after exposure to extracellular ATP via P2X7 receptors (Signal 2), and induces the activation of caspase-1, which leads to the secretion of IL-1β and pyroptosis. CS does not interfere with NF-κB signaling (Signal 1), but inhibits the activation of caspase-1 (Signal 2) by attenuating the adenosine monophosphate (AMP) kinase pathway, which is followed by the negative regulation of IL-1β secretion and pyroptosis.

This is the first study to show that cyclic stretch transduces an inhibitory signal specific for NLRP3 inflammasome signaling in LPS-primed murine monocyte-derived macrophages, which is a J774.1 macrophage cell line and BMDM. Cyclic stretch significantly suppressed ATP-induced IL-1β secretion as well as pyroptosis by inhibiting caspase-1 activity, partially through the suppression of AMPK activity, without affecting the NF-κB signaling pathway, as shown in **Figure 8**.

The role of cyclic stretch in the mechanisms of inflammation has remained controversial. Cyclic stretch was previously reported to activate NLRP3 inflammasomes in primary mouse and rat alveolar macrophages, leading to the activation of caspase-1 activity and increases in IL-1β secretion (Wu et al., 2013); however, it did not affect the production of TNF-α or IL-6 in rat alveolar macrophages (Lang et al., 2006). Furthermore, in rat peritoneal macrophages, cyclic stretch did not increase the expression of inflammatory genes, including IL-1β, IL-6, and Cox-2, whereas static stretch did (Wehner et al., 2010). Recent studies revealed that many tissue-resident macrophage populations, including those in the lungs, brain, and liver, arise from embryonic precursors, but not from blood monocytes (Lavin et al., 2015). The alveolar macrophage has been reported to self-maintain locally throughout adult life with minimal contributions from circulating monocytes (Guilliams et al., 2013; Hashimoto et al., 2013; Yona et al., 2013). The peritoneal macrophage has been shown to contain only a small subset of monocyte-derived macrophages in healthy tissues (Ghosn et al., 2010). Furthermore, tissue-resident macrophages are known to have tissue-specific phenotypes and functions, which may be regulated by tissue-specific factors, such as transcription factors (Gautier et al., 2012). In addition, macrophages in infiltrating in tumors have been reported to have potential pro-or anti-tumorigenic influence depending on the different tumor microenvironments (Papaccio et al., 2017). Thus, the different origins of macrophages may cause discrepancies in the effects of cyclic stretch on inflammatory responses. These discrepancies may also be caused in part by the magnitude and frequency of the mechanical stress and/or co-existing static stress, such as the stiffness of a culture dish on which surface cells adhere because the stiffness of the ECM/substrate is known to affect the macrophage activation phenotype and function (Patel et al., 2012; Previtera and Sengupta, 2015).

AMPK is a key regulator of cellular energy homeostasis (O’Neill and Hardie, 2013). It regulates not only cellular metabolism, but also cell survival and proliferation (Inoki et al., 2012). Recent studies reported that AMPK signaling is involved in ATP-induced inflammasome activation in LPS-primed macrophages, with the phosphorylated status of AMPK being enhanced by a stimulation with ATP (Liang et al., 2016; Zha et al., 2016). In the present study, we showed that AMPK signaling had a similar contribution to that reported previously using compound C, a specific inhibitor of AMPK, and also that cyclic stretch inhibited ATP-induced AMPK phosphorylation, suggesting that the cyclic stretch-mediated inhibition of IL-1β secretion was partially due to the suppression of AMPK signaling.

Specific molecules have been reported to regulate NLRP3 inflammasome signaling. The Cox-2-prostaglandin E_2_ axis positively regulates NLRP3 inflammasomes, leading to IL-1β secretion by J774A.1 cells (Hua et al., 2015) and BMDM (Zasłona et al., 2017). Tripartite-motif protein 30, which negatively regulates toll-like receptor (TLR)-mediated NF-κB activation by targeting the degradation of transforming growth factor β-activated kinase (TAK1)-binding protein (TAB)2-TAB3 (Shi et al., 2008), has been reported to inhibit NLRP3 inflammasome activation through the modulation of reactive oxygen species production in murine macrophages (Hu et al., 2010). Nuclear factor E2, which is an essential transcription factor mediating cellular redox homeostasis, is required for NLRP3 inflammasome activation (Zhao et al., 2014), and also inhibits NF-kB-dependent cytokine production in macrophages (Thimmulappa et al., 2006). In the present study, we clearly showed that cyclic stretch did not significantly affect the NF-kB signaling pathway, as evaluated using three parameters: IkBa degradation, NF-kB nuclear translocation, and NF-kB p65 transcriptional activity (**Figures 3A–D**). The independence of the NF-kB signaling pathway was also supported by our data (Supplemental Figure S1 and **Figure 4**) showing that cyclic stretch did not significantly alter the expression of Cox-2, proinflammatory cytokine production, or NLRP3 inflammasome components, which are NF-kB-dependent cellular responses (Tak and Firestein, 2001; Bauernfeind et al., 2009). Therefore, the mechanisms associated with the transcription factors described above do not appear to be involved in cyclic stretch-mediated inhibition.

Our results indicate that cyclic stretch plays an important role in preventing excessive inflammasome activation in mechanical environmental tissues (Oya et al., 2013; Mennens et al., 2017), such as the lungs, heart, arterial wall, and periodontal ligament, in which the infiltration of large numbers of macrophages derived from peripheral blood monocytes occurs during the processes of inflammation and tissue repair. Of these tissues, the periodontal ligament, which is located between the tooth root and its surrounding bone (Bartold, 2012), is subjected to mechanical stimuli, including cyclic stretch, from normal mastication. The lack of occlusal stimuli in rat models has been shown to induce atrophic changes with the up-regulation of IL-1β, while the down-regulation of IL-1β expression was observed when the occlusal state was restored using a customized occlusal plate, suggesting that cyclic occlusal stimuli regulate IL-1β expression (Boonpratham et al., 2007). Our results are consistent with these findings and also support the concept that the maintenance of periodontal health requires physiological mechanical stimuli (McCulloch et al., 2000).

In summary, although the activation of NLRP3 inflammasomes contributes to host defenses, the excessive activation of inflammasome signaling leads to pathological inflammation and tissue destruction. Therefore, mechanisms that tightly regulate NLRP3 inflammasomes are needed in order to maintain tissue homeostasis. Our results provide an insight into the maintenance of homeostasis through the prevention of excessive inflammasome activation.

## Ethics Statement

The experimental procedures were approved by the Ethical Review Board of Tohoku University Graduate School of Dentistry (Sendai, Japan).

## Author Contributions

KM and EN conceived, designed the study, and drafted the manuscript. KM and HT acquired the data. KM, YS, MS, HT, TN, SY, and EN analyzed and interpreted the data. EN finally approved the article.

## Conflict of Interest Statement

The authors declare that the research was conducted in the absence of any commercial or financial relationships that could be construed as a potential conflict of interest.
