# Supplementary figures and images for: Cyclic Stretch Negatively Regulates IL-1β Secretion Through the Inhibition of NLRP3 Inflammasome Activation by Attenuating the AMP Kinase Pathway
Source: Front Physiol. 2018 Jun 28;9:802. doi: 10.3389/fphys.2018.00802 (PMC6031751; doi:10.3389/fphys.2018.00802)

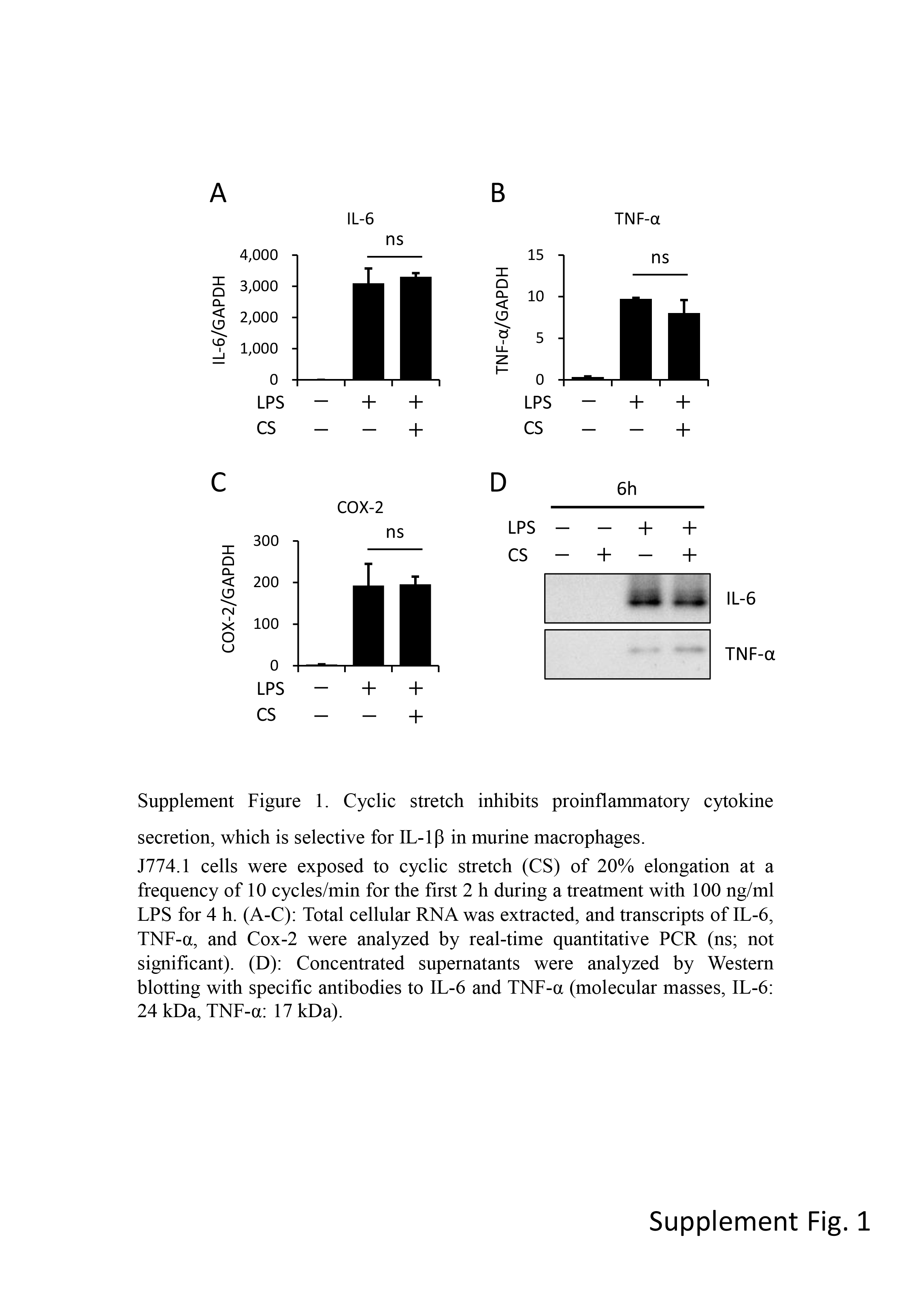

Supplement: Supplementary file 1 [file Image_1.TIF]

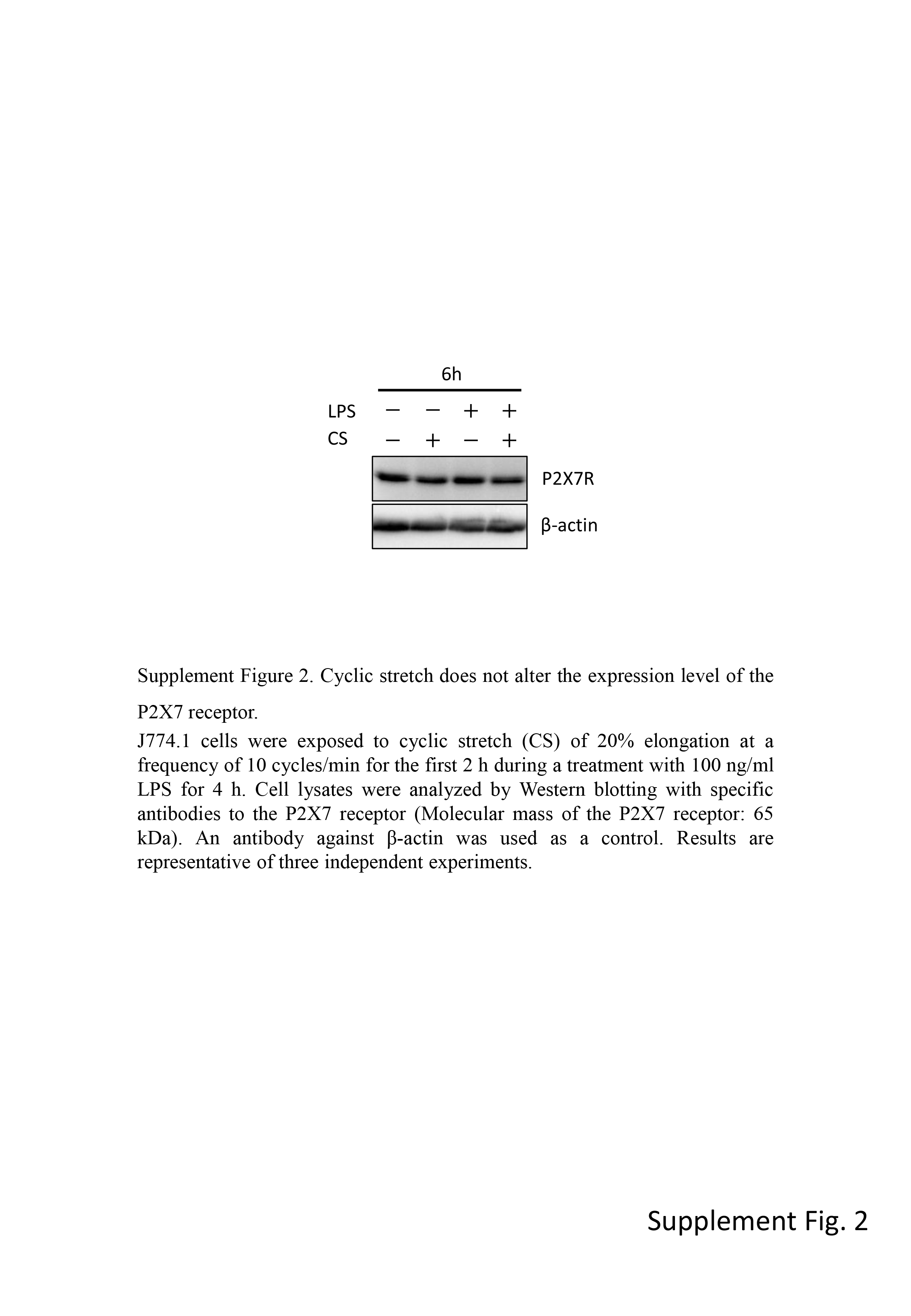

Supplement: Supplementary file 2 [file Image_2.TIF]
